# Insomnia as an independent predictor of suicide attempts: a nationwide population-based retrospective cohort study

**DOI:** 10.1186/s12888-018-1702-2

**Published:** 2018-05-02

**Authors:** Han-Ting Lin, Chi-Huang Lai, Huey-Jen Perng, Chi-Hsiang Chung, Chung-Ching Wang, Wei-Liang Chen, Wu-Chien Chien

**Affiliations:** 1 National Defense Medical Center, School of Public Health 4325R, No. 161, Section 6, Min-Chuan East Road, Neihu District, Taipei City, 11490 Taiwan, Republic of China; 2 National Defense Medical Center, Graduate Institute of Life Sciences 7115R, No. 161, Section 6, Min-Chuan East Road, Neihu District, Taipei City, 11490 Taiwan, Republic of China; 3 Department of Family and Community Medicine, National Defense Medical Center, Tri-Service General Hospital Taipei, No. 325, Section 2, Cheng-Kung Road, Taipei City, 11490 Taiwan, Republic of China; 4 Department of Medical Research 7115R, National Defense Medical Center, Tri-Service General Hospital Taipei, No. 325, Section 2, Cheng-Kung Road, Taipei City, 11490 Taiwan, Republic of China

**Keywords:** Insomnia, Suicide, National Health Insurance Research Database (NHIRD)

## Abstract

**Background:**

Numerous studies have verified that insomnia is associated with suicidal ideation, suicide attempts, and death by suicide. Limited population-based cohort studies have been conducted to examine the association. The present study aimed to analyze whether insomnia increases the risk of suicide attempts and verify the effects of insomnia on suicide risk.

**Methods:**

This study is a cohort study using 2000–2013 hospitalization data from the National Health Insurance Research Database (NHIRD) to track the rate of suicide attempts among insomnia patients aged 15 years or older. In addition, a 1:2 pairing based on sex, age, and date of hospitalization was conducted to identify the reference cohort (patients without insomnia). Cox proportional hazard model was used to assess the effects of insomnia on suicide risk.

**Results:**

The total number of hospitalized patients aged 15 years or older was 479,967 between 2000 and 2013 (159,989 patients with insomnia and 319,978 patients without insomnia). After adjusting for confounders, suicide risk in insomnia patients was 3.533-fold that of patients without insomnia (adjusted hazard ratio [HR] = 3.533, 95% confidence interval [CI] = 3.059–4.080, *P* < 0.001). Suicide risk in low-income patients was 1.434-fold (adjusted HR = 1.434, 95% CI = 1.184–1.736, *P* < 0.001) that of non-low-income patients. Suicide risk in patients with drug dependence and with mental disorders was 1.592-fold (adjusted HR = 1.592, 95% CI = 1.220–2.077, *P* < 0.001) and 4.483-fold (adjusted HR = 4.483, 95% CI = 3.934–5.109, *P* < 0.001) that of patients without drug dependence and without mental disorders, respectively. In the female population, suicide risk in insomnia patients was 4.186-fold (adjusted HR = 4.186, 95% CI = 3.429–5.111, *P* < 0.001) that of patients without insomnia. Among patients aged 25–44 years, suicide risk in insomnia patients was 5.546-fold (adjusted HR = 5.546, 95% CI = 4.236–7.262, *P* < 0.001) that of patients without insomnia. Furthermore, the suicide risk of insomnia patients with mental disorders was 18.322-fold that of patients without insomnia and mental disorders (*P* < 0.001).

**Conclusion:**

Insomnia, low income, drug dependence, and mental disorders are independent risk factors for suicide attempts. Female patients and those aged 25–44 years are at high risk of suicide due to insomnia. Insomnia, mental disorders, and low income exhibit a synergistic effect on suicide attempts. Clinicians should pay attention to mental status and income level of insomnia patients.

**Electronic supplementary material:**

The online version of this article (10.1186/s12888-018-1702-2) contains supplementary material, which is available to authorized users.

## Background

Suicide is a serious public health problem around the world. The World Health Organization (WHO) reported that approximately 800,000 people worldwide die from suicide every year (an average of one death every 40 s). In addition, suicide is the second leading cause of death among those aged 15–29 years globally, and the fifth leading cause of death among those aged 30–49 years [1]. Compared with death by suicide, there are many more suicide attempts every year [2], and according to a meta-analysis from Japan in 2008, a prior suicide attempt is the most important predictor of suicide [3]. Suicide, moreover, causes immense socioeconomic burdens on families, communities, and nations [4]. In 2016, the number of deaths from suicide in Taiwan was 3765 (a suicide death rate of 16.0 persons per 100,000 population), making suicide the twelfth leading cause of death in Taiwan. The suicide death rate among men was approximately 2.1-times higher than that among women (17.0 men and 7.7 women per 100,000 population). The suicide death rate in all age groups increased with age [5].

Insomnia is one of the most prevalent sleeping disorders in the world [6]. According to epidemiological studies around the world, the prevalence of insomnia in general populations is 10%–25% [7–10]. A 2017 report by the Taiwan Society of Sleep Medicine indicated that a tenth of the population across Taiwan suffers from chronic insomnia (prevalence rate of 11.3%) [11]. Insomnia refers to difficulty falling asleep, remaining awake while trying to sleep, waking up often during the night, or still feeling tired after sleeping for a brief period, factors that subsequently influence daytime activities of daily living for more than four weeks. If insomnia persists for more than six months, it becomes a chronic condition influencing not only the person’s psychology and physiology but also his or her health and activities of daily living (e.g., learning and working) [12].

Numerous studies have indicated that insomnia is associated with suicidal ideation [13–16], suicide attempts [17–19], and death by suicide [20–22] among adolescents and adults. These studies laid the foundation for the relationship between insomnia and suicide. However, there are some weakness in methodology and need future research to fill this gap. For example, some studies used a questionnaire asking a single question as criteria for determining insomnia or suicide [22]. Furthermore, mental disorders are a major confounding factor for insomnia and suicide, and over 90% of suicide decedents have at least one mental disorder [23]; however, some studies assessing the link between insomnia and suicide did not adjust for mental disorders [17, 20].

There are still different arguments about whether insomnia is an independent factor of suicide or if insomnia, as a symptom of mental disorders such as depression, increases the risk of suicide. Some studies have found that the relationship between insomnia and suicide is fully mediated by mental disorders [13, 14, 24]. Other studies have shown that insomnia remains an independent factor of suicidal ideation [15, 16], suicide attempt [19], and death by suicide [21, 22] after adjustments for mental disorders, substance abuse and alcohol abuse.

A meta-analysis by Pigeon et al. indicated that insomnia is still a predictor of suicide even after other factors have been adjusted for. However, the authors asserted that the samples collected are non-homogeneous, and samples for observational and clinical studies are confined to adolescents or adults only. Additionally, numerous studies are cross-sectional studies [25], which cannot be used to elucidate whether insomnia increases suicide risk. Hence, we employed the National Health Insurance Research Database (NHIRD) in Taiwan to conduct a retrospective cohort study of whether insomnia increases the risk of suicide attempts.

## Methods

### Data source

Introduced in Taiwan in 1995, the NHRID contains the medical records of all insured. The database encompasses the information of more than 99% of the 23 million people who live in Taiwan; thus, the health care information contained therein represents evidence-based data in the medicine and healthcare sector [26]. This study analyzed the 2000–2013 inpatient expenditure files and the registry for contracted medical facilities in the NHRID.

#### Design and sample

The retrospective cohort study design method was adopted in this study. Inpatients aged 15 years or older who were newly diagnosed with insomnia (International.

Classification of Diseases, 9th Revision, Clinical Modifications [ICD-9-CM]: 307.41, 307.42, 780.52) were allocated to the study cohort. To ensure that the patients were newly diagnosed, we also excluded patients who were diagnosed with insomnia between 1997 and 1999. The study period began on January 1, 2000 and ended on December 31, 2013. In total, 159,989 participants were included. The average of follow-up for the diagnosis of insomnia was 6.47 years. To facilitate comparison of the study cohort, propensity score-matching based on sex, age group, and date of hospitalization was performed for a 1:2 pairing to identify the reference cohort (*n* = 319,978 inpatients who were diagnosed as not having insomnia). Furthermore, there was no difference in the cause of hospitalization at baseline (Additional file 1: Table S1).

#### Outcome assessment

All research participants were observed until the incidence of suicide attempts (ICD-9-CM E code: 950–959), loss to follow up, or until the end of December 31, 2013.

#### Covariates of interested

The participants in this study were allocated into four groups by age: 15–24, 25–44, 45–64, and ≥ 65 years. Urbanization levels were categorized into high, moderate, and low levels of urbanization [27]. Low income included insured individuals in Category 5 (those to which laws and regulations governing social relief apply). Catastrophic illness was indicated in the copayment remarks of inpatient expenditure files. The Charlson comorbidity index (CCI) was used to weigh the 19 types of diseases by assigning each with a score of 1, 2, or 6, and the scores were summed according to whether each patient had any of these diseases [28, 29]. Because drug dependence, alcohol dependence, and mental disorders are risk factors for suicide attempts, drug dependence (ICD-9-CM 292, 304), alcohol dependence (ICD-9-CM 291, 303), and mental disorders (ICD-9-CM 290–319, excluding 291, 292, 303, 304) were incorporated in the regression model for adjustments.

#### Statistical analysis

All analyses were performed using SPSS 22 (SPSS, Inc., Chicago, IL, U.S.). The χ^2^ test and Fisher exact test were used to compare the categorical variables of the two groups, and a t-test was conducted to compare the continuous variables of the two groups. With adjustments for sex, age, low income, catastrophic illness, urbanization, CCI, drug dependence, alcohol dependence, and mental disorders, Cox proportional hazards regression analyses were performed to assess the effects of insomnia on the risk of suicide attempts and presented as hazard ratio (HR) with a 95% confidence interval (CI). Furthermore, differences in the risk of suicide attempts between the study and reference cohort were estimated using the Kaplan-Meier method with the log-rank test. A 2-tailed *p*-value < 0.05 was considered to indicate statistical significance.

## Results

**Fig. 1:**
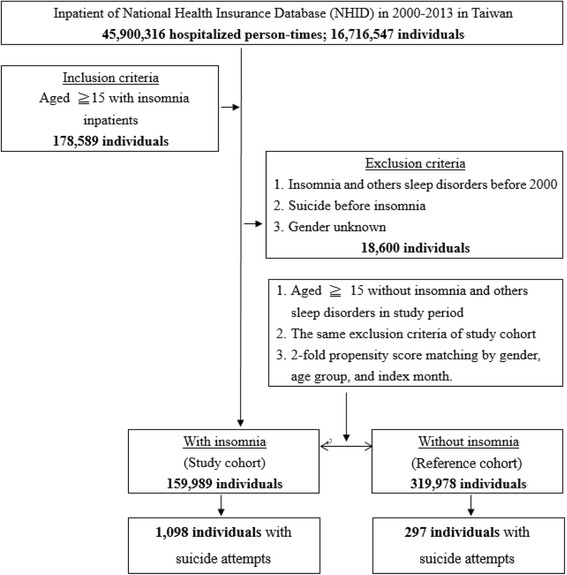
Flowchart of study sample selection from the National Health Insurance Research Database in Taiwan

**Fig. 2:**
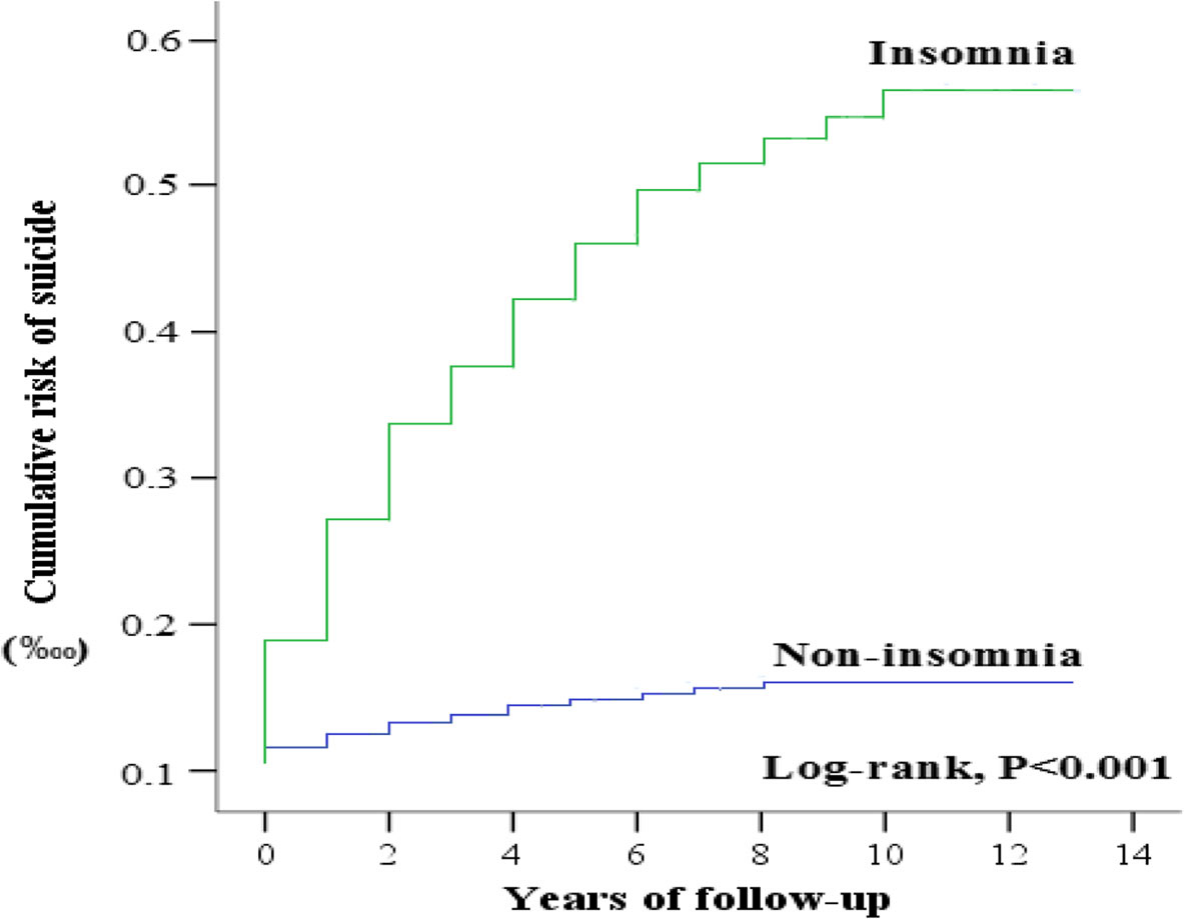
Kaplan-Meier analysis of the cumulative risk of suicide attempts in 13 years of tracking stratified by insomnia with log-rank test

Figure 1 shows the case-screening process (inclusion and exclusion) and follow-up results as well as the cumulative risk of suicide incidence between the two groups (patients with/without insomnia). Between 2000 and 2013, 16,716,547 patients were hospitalized, 178,589 of which had insomnia. After exclusion criteria were applied (18,600 excluded), there were a total of 159,989 insomnia cases (319,978 patients were placed in the reference cohort based on 1:2 pairing) with a subsequent suicide incidence of 0.69% (1098/159,989), whereas the reference cohort exhibited a suicide incidence of 0.09% (297/319,978). The groups demonstrated significant difference (log-rank *P* < 0.001). In other words, the probability (risk) of suicide attempts in insomnia patients was considerably higher than that in patients without insomnia. In addition, both groups exhibited a statistically significant difference after one year follow up (Fig. 2, Table 1). Furthermore, an average follow-up period for the diagnosis of insomnia to suicide attempts was 2.38 years.

**Table 1:** Kaplan-Meier analysis of the cumulative risk of suicide attempts in 13 years of tracking stratified by insomnia with log-rank test

Insomnia	With (*N* = 159,989)	Without (*N* = 319,979)	*P*-value
*X*-year(s) of suicide attempts	Numbers of suicide attempts		
1	285	101	< 0.001
2	525	157	< 0.001
3	700	195	< 0.001
4	808	222	< 0.001
5	890	248	< 0.001
6	965	265	< 0.001
7	1020	276	< 0.001
8	1052	286	0.001
9	1070	295	< 0.001
10	1084	296	< 0.001
11	1198	297	< 0.001
12	1198	297	< 0.001
13	1198	297	< 0.001

Table 2 presents the basic characteristics of the 479,967 patients at the endpoint of the follow-up process (study cohort = 159,989 insomnia patients; reference cohort = 319,978 non-insomnia patients). The study cohort exhibited a substantially higher incidence of suicide attempts compared with the reference cohort (0.7% vs 0.1%; *P* < 0.001). The study cohort comprised significantly higher numbers of low income patients (4.0% vs 1.5%; *P* < 0.001), patients with catastrophic illness (31.2% vs 19.8%; *P* < 0.001), and those who lived in working class urbanized townships (33.4% vs 22.3%; *P* < 0.001) than the reference cohort. The numbers of patients with drug dependence (1.0% vs 0.1%; *P* < 0.001), alcohol dependence (3.5% vs 0.7%; *p* < 0.001), and mental disorders (33.5% vs 7.1%; *p* < 0.001) and other comorbidities in the study cohort were significantly higher than those of the reference cohort.

**Table 2:** Characteristics of study in the endpoint

	Total		With insomnia		Without insomnia		*P*-value
Variables	n	%	n	%	n	%	
Total	479,967	100.0	159,989	33.3	319,978	66.7	
Suicide attempts							< 0.001
with	1395	0.3	1098	0.7	297	0.1	
without	478,572	99.7	158,891	99.3	319,681	99.9	
Gender							0.880
male	235,771	49.1	78,615	49.1	157,156	49.1	
female	244,196	50.9	81,374	50.9	162,822	50.9	
Age group (years)							0.958
15–24	15,901	3.3	5330	3.3	10,571	3.3	
25–44	99,058	20.6	33,030	20.6	66,028	20.6	
45–64	172,650	36.0	57,504	35.9	115,146	36.0	
≧65	192,358	40.1	64,125	40.1	128,233	40.1	
Low income							< 0.001
yes	11,251	2.3	6441	4.0	4810	1.5	
no	468,716	97.7	153,548	96.0	315,168	98.5	
Catastrophic illness							< 0.001
yes	113,150	23.6	49,914	31.2	63,236	19.8	
no	366,817	76.4	110,075	68.8	256,742	80.2	
Urbanization							< 0.001
high	150,124	31.3	41,562	26.0	108,562	33.9	
medium	205,154	42.7	65,049	40.7	140,105	43.8	
low	124,689	26.0	53,378	33.4	71,311	22.3	
CCI	2.36 ± 3.82		2.97 ± 4.10		2.06 ± 3.63		< 0.001
Drug dependence							< 0.001
yes	2051	0.4	1597	1.0	454	0.1	
no	477,916	99.6	158,392	99.0	319,524	99.9	
Alcohol dependence							< 0.001
yes	7846	1.6	5674	3.5	2172	0.7	
no	472,121	98.4	154,315	96.5	317,806	99.3	
Mental disorders							< 0.001
yes	76,322	15.9	53,537	33.5	22,785	7.1	
no	403,645	84.1	106,452	66.5	297,193	92.9	

*P*-value (category variable: Chi-square/Fisher exact test; continue variable: t-test)

Table 3 shows the univariate and multivariate analysis results of the factors of suicide attempts. After the research variables (sex, age, low income, catastrophic illness, urbanization, CCI, drug dependence, alcohol dependence, and mental disorders) were adjusted for, the risk of suicide attempts among insomnia patients was 3.533-fold that of non-insomnia patients (*P* < 0.001). The risk of suicide attempts among female patients was 1.522-fold (*P* < 0.001) that of male patients. The risk of suicide attempts among patients aged 15–24, 25–44, 45–64 years was 6.000-fold, 3.581-fold, and 1.595-fold (*P* < 0.001) that of patients aged 65 years or older, respectively. The risk of suicide attempts among low-income patients and patients with catastrophic illness was 1.434-fold and 1.286-fold that of their counterparts, respectively (*P* < 0.001). The risk of suicide attempts among patients with drug dependence and mental disorders was 1.592-fold and 4.483-fold that of their counterparts, respectively (*P* < 0.001).

**Table 3:** Factors of suicide attempts by Cox proportional hazard model analysis

Variables	Crude HR	95% CI		*P*-value	Adjusted HR	95% CI		*P*-value
Insomnia								
no	reference				reference			
yes	7.416	6.524	8.431	< 0.001	3.533	3.059	4.080	< 0.001
Gender								
female	reference				reference			
male	1.289	1.159	1.433	< 0.001	1.522	1.361	1.702	< 0.001
Age group (years)								
≧65	reference				reference			
15–24	6.477	5.292	7.928	0.001	6.000	4.876	7.383	< 0.001
25–44	4.909	4.238	5.687	< 0.001	3.581	3.069	4.177	< 0.001
45–64	1.747	1.487	2.052	< 0.001	1.595	1.357	1.876	< 0.001
Low income								
no	reference				reference			
yes	3.940	3.267	4.751	< 0.001	1.434	1.184	1.736	< 0.001
Catastrophic illness								
No	reference				reference			
Yes	1.972	1.771	2.196	< 0.001	1.286	1.143	1.446	< 0.001
Urbanization								
high	reference				reference			
medium	1.045	0.916	1.193	0.510	1.013	0.887	1.157	0.847
low	1.569	1.372	1.795	< 0.001	1.073	0.937	1.230	0.309
CCI	0.983	0.969	0.998	0.025	0.989	0.973	1.005	0.179
Drug dependence								
no	reference				reference			
yes	9.614	7.407	12.479	< 0.001	1.592	1.220	2.077	0.001
Alcohol dependence								
no	reference				reference			
yes	5.872	4.899	7.038	< 0.001	1.164	0.955	1.420	0.133
Mental disorders								
no	reference				reference			
yes	9.742	8.723	10.879	< 0.001	4.483	3.934	5.109	< 0.001

Table 4 shows the hierarchical analysis of various variables to elucidate the difference in risk of suicide attempts between insomnia and non-insomnia patients. The results indicated that (after other factors were adjusted for), in the female population, suicide risk in insomnia patients was 4.186-fold (adjusted HR = 4.186, 95% CI = 3.429–5.111, *P* < 0.001) that of patients without insomnia. Among patients aged 25–44 years, suicide risk in insomnia patients was 5.546-fold (adjusted HR = 5.546, 95% CI = 4.236–7.262, *P* < 0.001) that of patients without insomnia.

**Table 4:** Factors of suicide attempts stratified by variables listed in the table by Cox proportional hazard model analysis

Variables	With insomnia			Without insomnia			Ratio	Adjusted HR	95%CI		*P*-value
	Event	PYs	Rate (per 10^3^ PYs)	Event	PYs	Rate (per 10^3^ PYs)					
Total	1098	953,594	115.143	297	1,913,519	15.521	7.418	3.533	3.059	4.080	< 0.001
Gender											
female	633	468,954	134.981	153	941,910	16.244	8.310	4.186	3.429	5.111	< 0.001
male	465	484,640	95.948	144	971,609	14.821	6.474	2.861	2.323	3.523	< 0.001
Age group (years)											
15–24	119	42,625	279.179	33	85,435	38.626	7.228	2.778	1.747	4.417	< 0.001
25–44	546	198,523	275.031	80	400,512	19.974	13.769	5.546	4.236	7.262	< 0.001
45–64	264	317,825	83.065	105	637,466	16.471	5.043	2.637	2.053	3.389	< 0.001
≧65	169	394,621	42.826	79	790,106	9.999	4.283	2.900	2.188	3.844	< 0.001
Low income											
no	987	915,626	107.795	288	1,884,404	15.283	7.053	3.487	3.009	4.040	< 0.001
yes	111	37,968	292.351	9	29,115	30.912	9.458	4.069	2.012	8.228	< 0.001
Catastrophic illness											
no	635	645,939	98.306	220	1,504,259	14.625	6.722	3.769	3.171	4.479	< 0.001
yes	463	307,655	150.493	77	409,260	18.814	7.999	2.916	2.255	3.770	< 0.001
Urbanization											
high	291	243,831	119.345	84	661,398	12.700	9.397	3.911	2.964	5.161	< 0.001
medium	398	369,572	107.692	132	835,185	15.805	6.814	3.320	2.655	4.153	< 0.001
low	409	340,191	120.227	81	416,936	19.427	6.188	3.355	2.592	4.342	< 0.001
Drug dependence											
no	1044	942,877	110.725	292	1,910,198	15.286	7.243	3.569	3.086	4.127	< 0.001
yes	54	10,717	503.872	5	3321	150.557	3.347	2.079	0.817	5.286	0.207
Alcohol dependence											
no	978	916,596	106.699	288	1,899,319	15.163	7.037	3.513	3.032	4.072	< 0.001
yes	120	36,998	324.342	9	14,200	63.380	5.117	3.844	1.936	7.634	< 0.001
Mental disorders											
no	300	618,043	48.540	181	1,763,589	10.263	4.730	4.798	3.968	5.801	< 0.001
yes	798	335,551	237.818	116	149,930	77.369	3.074	2.223	1.823	2.711	< 0.001

**Fig. 3:**
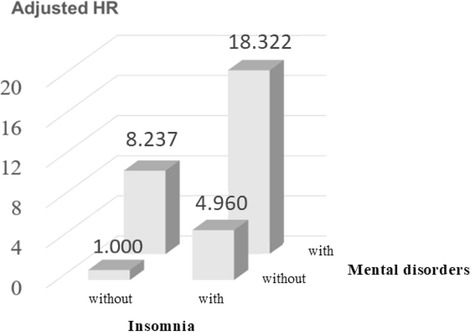
Interaction for risk of suicide attempts by insomnia and mental disorders

**Fig. 4:**
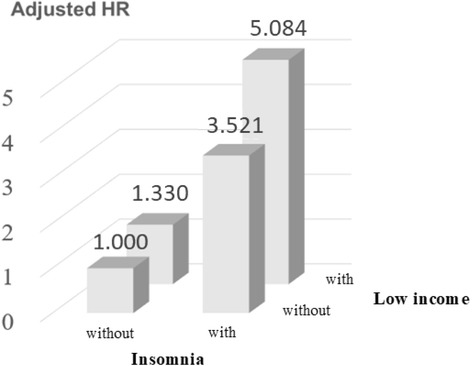
Interaction for risk of suicide attempts by insomnia and low income

Figures 3 and 4 show the interactive effects of insomnia, mental disorders, and low income after other factors were adjusted for. The risk of suicide attempts in insomnia patients without mental disorders was 4.960-fold that of patients without insomnia and mental disorders. The risk of suicide attempts in non-insomnia patients with mental disorders was 8.237-fold that of patients without insomnia and mental disorders. The risk of suicide attempts in insomnia patients with mental disorders was 18.322-fold that of patients without insomnia and mental disorders (*P* < 0.001) (Fig. 3). The risk of suicide attempts in non-low-income patients with insomnia was 3.521-fold that of non-insomnia patients who were not in the low-income group. The risk of suicide attempts in low-income patients without insomnia was 1.330-fold that of non-insomnia patients who were not in the low-income group. The risk of suicide attempts in low-income patients with insomnia was 5.084-fold that of non-insomnia patients who were not in the low-income group (*P* < 0.001) (Fig. 4).

## Discussion

Our study found that insomnia remained an independent risk factor for suicide attempts after adjustments for mental disorders, alcohol dependence, and drug dependence. To the best of our knowledge, our study is the first retrospective cohort study to use population-based data and clinical diagnosis as the criteria for determining insomnia and suicide. The findings can reinforce the deficiencies of other relevant studies.

In recent years, some studies found that mental disorders, alcohol abuse, and drug abuse do not fully mediate the association between insomnia and suicide. A study conducted in 2012 regarding members of the military in the United States reported that insomnia was an independent risk factor for suicidal ideation after adjustments for depression, hopelessness, post-traumatic stress disorder, anxiety, drug abuse, and alcohol abuse. However, there were no significant associations between insomnia and suicide attempts [15]. Different from the above study [15] using a 3-item questionnaire as the criteria for insomnia, our study used the clinical diagnosis as the criteria for determining insomnia. Another study conducted in the United States in 2016 revealed that insomnia symptoms increase the risk of suicidal ideation and attempts in adolescents with adjustments for mental disorders and substance use disorders [19]. However, the above study [19] had several limitations. First, the study was cross-sectional. No temporal relationships could be established. Second, all measures were based on self-report, thus the data were subject to response and recall bias. Therefore, based on above study [19], we established insomnia as an independent predictor of suicide attempts.

Our study found that insomnia and mental disorders have a synergistic effect on the risk of suicide attempts. The risk of suicide attempts in insomnia patients with mental disorders was 18-fold that of patients without insomnia and mental disorders. However, no comparison with past studies can be made because investigations concerning the interactive effects of insomnia and mental disorders on suicide attempts are lacking. A cross-sectional study conducted in 2018 in the United States reported that poor sleep quality will increase the risk of suicide after adjusting for depression among adolescents. However, sleep problems and depression do not interact with the risk of suicide [30]. Different from above study [30] focus on the association between sleep problems and suicide, our study focused on insomnia and suicide attempts. Therefore, it cannot be compared.

The mechanisms underlying the relationship between insomnia and suicide remain unclear. In 2013, a systematic literature review of adult insomnia and suicide conducted in the United States indicated that the mechanisms of insomnia and suicide involve both physiological and psychological dimensions [31]. The physiological mechanism includes a reduction in serotonin [32] and hypothalamic-pituitary-adrenal (HPA) axis dysfunction [33]. The psychological mechanism is associated with dysfunctional beliefs and attitudes about sleep (DBAS) [15] and depression [34]. Furthermore, a previous study found that fatigue resulting from sleep disorders may lead to hopelessness and decrease impulse control, both demonstrated risk factors for suicide [35]. The above possible mechanisms between insomnia and suicide require further studies for confirmation.

Our study determined that the risk of suicide attempts in patients with drug dependence was 1.592-fold that of patients without drug dependence. Several studies have indicated that the abuse of drugs such as marijuana [36], heroin [37, 38], and nicotine [39] are risk factors for suicide. A review article reported that 25% to 50% of people who are suicidal are dependent on alcohol or drugs, and the risk of suicide increases if these people have mental disorders [40]. A study conducted in 1995 regarding indigenous peoples in Taiwan reported that the risk of suicide in people with substance dependence and depression was 470.2-fold that of people without substance dependence and depression [41]. In our study, we found that the risk of suicide attempts in patients with both substance dependence and depression was 22.7-fold that of their counterparts who did not fact these issues, which is lower than the finding of the aforementioned study. This difference might be attributable to social group, population, and cultural discrepancies. Another study conducted in the United States in 1993 revealed that the risk of suicide in patients with substance dependence and an emotional disorder was 17.0-fold that of their counterparts [42]. By contrast, we determined that the risk of suicide attempts in patients with both substance dependence and emotional disorder was 23.4-fold that of their counterparts. Similar to the Taiwan study [41], the focus of substance abuse in this study was alcohol and drugs; however, we reported a lower rate of substance abuse (alcohol abuse = 9.2% and drug abuse = 4.3%) compared with that of [41] (alcohol abuse = 24.3% and drug abuse = 13.4%). This difference is possibly attributed to the more stringent standard of substance abuse (individuals exhibiting substance dependence) adopted in the present study.

Our study found that the risk of suicide attempts in patients with mental disorders was 4.483-fold that of patients without mental disorders. In western countries such as the United Kingdom, a study reported that 90% of suicides were associated with a history of mental disorders [43]. In Asian countries such as China, 40% to 70% of suicides were associated with a history of mental disorders [44, 45]. Multiple studies have indicated that different types of mental disorders predict different levels of suicide risk [46–49]. In particular, schizophrenia has the highest risk for suicide [50]. In our study, depression had the highest risk for suicide attempts, followed by schizophrenia. Another study revealed that the risk of suicide in patients with more than one mental disorders was 1.5–2.5-fold that of patients with only one type of mental disorders [43]. We incorporated depression, anxiety, bipolar disorder, and schizophrenia for further analysis and found that for every increase in the number of mental disorders, the risk of suicide attempts increased by 0.6-fold. This finding is somewhat similar to that reported in the aforementioned study.

In 2016, a United Kingdom systematic literature review indicated that low income, low socioeconomic status, and unemployment predicted higher levels of suicidal ideation, suicidal behavior, and death by suicide [51]. Our study found that risk of suicide attempts in low-income patients was 1.434-fold that of their counterparts (adjusted HR = 1.431, 95% CI = 1.182–1.732, *P* < 0.001). Furthermore, the risk of suicide attempts in insomnia patients who earn low levels of income was 4.960-fold that of their counterparts (*P* < 0.001). This result implies that under the interactive effects of an economic stressor (low income) and insomnia, suicide became a major option for this particular population (particularly women, people aged 25–44 years, and breadwinners in the family), thereby leading to regrettable circumstances.

Our study has the following limitations. First, due to the limited types of data provided in the NHIRD, we were unable to acquire important suicide-related information (e.g., stigma, family history of suicide, occurrence of material events, and network of support). Second, our study adopted inpatient expenditure files from the NHIRD for analysis; therefore, our study findings may not be generalizable to the entire population without including outpatient cases. Third, all information was collected from medical record (NHIRD). The possibility of information bias cannot be ruled out.

## Conclusions

Insomnia is a risk factor for suicide attempts and, indeed, increases the risk for suicide. In addition, low income, drug dependence, and mental disorders are risk factors for suicide attempts. Female patients and those aged 25–44 years are high-risk groups. Risk of suicide attempts is higher in low-income individuals with insomnia and mental disorders. Clinicians should pay attention to the mental status and income level of insomnia patients and implement early suicide prevention intervention. If members of the general public have friends who suffer from insomnia, they should pay attention to the mental and financial status of their friends in order to reduce the probability of suicide.

## Additional file

Additional file 1: **Table S1.** Main diagnosis for hospitalization in the baseline. (DOCX 15 kb)

## Abbreviations

CCI: The Charlson comorbidity index
CI: Confidence interval
HR: Hazard ratio
ICD-9-CM: The International Classification of Diseases, 9th Revision, Clinical Modifications
NHIRD: National Health Insurance Research Database
WHO: World Health Organization
