# Supplementary material for: Insomnia as an independent predictor of suicide attempts: a nationwide population-based retrospective cohort study
Source: BMC Psychiatry. 2018 May 2;18:117. doi: 10.1186/s12888-018-1702-2 (PMC5930777; doi:10.1186/s12888-018-1702-2)
Supplement: Supplementary file 1 — Table S1. Main diagnosis for hospitalization in the baseline. (DOCX 15 kb) [file 12888_2018_1702_MOESM1_ESM.docx]

| **Table S1.** Main diagnosis for hospitalization in the baseline | | | | |
| --- | --- | --- | --- | --- |
| **Variables** | **Insomnia** | | **Non-insomnia** | |
|  | **N=159,989** | | **N=319,978** | |
|  | **n** | **%** | **n** | **%** |
| **Main diagnosis for hospitalization** |  |  |  |  |
| Diseases of the circulatory system | 24,230 | 15.1 | 47,246 | 14.8 |
| Diseases of the digestive system | 22,761 | 14.2 | 46,579 | 14.6 |
| Diseases of the respiratory system | 20,341 | 12.7 | 32,316 | 10.1 |
| Neoplasms | 13,629 | 8.5 | 35,770 | 11.2 |
| Diseases of the nervous system and sense organs | 8,247 | 5.2 | 16,116 | 5 |
| **Total** | 89,208 | 55.8 | 178,027 | 55.6 |
